# Loot boxes use, video gaming, and gambling in adolescents: Results from a path analysis before and during COVID-19-pandemic-related lockdown in Italy

**DOI:** 10.3389/fpsyg.2022.1009129

**Published:** 2022-09-27

**Authors:** Caterina Primi, Francesco Sanson, Marta Vecchiato, Emilia Serra, Maria Anna Donati

**Affiliations:** ^1^NEUROFARBA Department, University of Florence, Florence, Italy; ^2^Addictions Department, Azienda ULSS n. 4 Veneto Orientale, Venice, Italy

**Keywords:** loot boxes, video gaming, gambling, adolescents, path analysis, COVID-19 pandemic, prevention, treatment

## Abstract

Loot Boxes (LBs), i.e., virtual items embedded within video games with numerous features reminiscent of gambling, are increasingly widespread among adolescents. LB use is associated with problem gambling in youth, but few studies have been conducted on the association between LB use and gambling behavior considering adolescents. Thus, the mechanisms underlying this relationship are not clear. As LB use is a significant and positive risk factor for video gaming severity, and video gaming is associated with problem gambling, we hypothesized that LB use would be related to gambling frequency and problem gambling through the intermediary role of video gaming frequency and problem video gaming. We tested our hypothesis before COVID-19-pandemic-related lockdown and during COVID-19-pandemic-related lockdown, in Italy. Study 1 was conducted with 1,078 high school students (39% boys, mean age = 16.46, *SD* = 1.28) before COVID-19-pandemic-related lockdown, and Study 2 was conducted with 1,204 high school students (57% boys, mean age = 15.62, *SD* = 0.64) during COVID-19-pandemic-related lockdown. A path analysis was carried out to analyze the significance of indirect effects from LB use to gambling behavior and problem gambling through video gaming and problem video gaming. In both the studies, results showed that LB use exerted significant and positive direct effects on video gaming frequency, problem video gaming, and gambling frequency. Moreover, LB use had indirect significant and positive effects on problem gambling through video gaming frequency, problem video gaming, and gambling frequency. Findings attest that LB use can be related to problem gambling through a greater involvement in videogames. Thus, a specific subtype of young gamblers can develop Gambling Disorder symptoms from the use of LBs and through an intense use of video games. Practical implications for prevention and treatment are discussed.

## Introduction

Loot Boxes (LBs) can be defined as virtual items embedded within video games that players can open with real-world money to get the chance of obtaining one or more virtual rewards. One of the main characteristics of LBs is that the reward is subjected to chance; therefore, players do not know which virtual reward they will get from their purchases. Features of LBs vary across games. Some of them give advantages in multiplayer games as reward (Drummond and Sauer, 2018), while others contain cosmetic items (for a full description of LBs’ features, see Zendle et al., 2019). LBs have become increasingly prevalent in video games (Zendle et al., 2019), with the most widespread games in the world that currently include LBs. Indeed, the presence of LBs in videogames has had a 67% increase between 2010 and 2019 (Zendle et al., 2020), with the rates of LBs adult purchasers of 44% (Li et al., 2019) and 78% (Zendle and Cairns, 2018). The use of the LBs has become increasingly even with adolescents. In a recent review (Montiel et al., 2022), the prevalence of LBs among adolescents has been specifically analyzed. Only four out of the 16 selected studies regarded youth with 12–18 years of age (Zendle et al., 2019; Kristiansen and Severin, 2020; DeCamp, 2021; Ide et al., 2021). With a sample of Delawarean adolescents, DeCamp (2021) found an annual prevalence of LBs purchases that ranged from 17% for ages 16%–17% to 24.9% for ages 13–14. These percentages become 28.3% and 33.9%, respectively, considering only adolescents who play video games. The annual prevalence rate of LBs purchasing among young gamers was 20% in Danish adolescents between 12 and 17 years of age (Kristiansen and Severin, 2020). In the United States of America, Zendle et al. (2019) analyzed the monthly prevalence of LBs purchases in adolescent gamers (aged 16–18 years) and reported it to be 40.5%, while, in Japan, Ide et al. (2021) found a purchase prevalence of 3.5% in young gamers of 14 years old.

Money transactions and the chance-based nature of LBs have led researchers to examine if LBs can represent a form of gambling, that can be defined as an activity that takes place when an item of value, usually money, is staked on the outcome of an event that is, to some degree, unpredictable (Ladouceur and Ferland, 2003). In this regard, King and Delfabbro (2018, p. 113) stated that LBs are like slot machines “*because they require no player skill and have a randomly determined outcome*.” This similarity was confirmed by Drummond and Sauer (2018), who systematically analyzed 22 of the most popular video games released with LBs in 2016–2017. Results showed that 10 of the 22 video games had LBs that met all five gambling characteristics according to Griffiths' criteria (1995), i.e., (I) the exchange of money or item of value; (II) exchange is determined by an event with uncertain outcome; (III) the event is at least partially determined by chance; (IV) losses can be avoided through non-participation; and (V) gambling revenues are made of money/items lost by players.

Gambling activities, such as sport bets, scratch cards, or cards for money, are widely spread among adolescents with 60% or more of them that wagered at least once a year (Calado et al., 2017; Pérez-Albéniz et al., 2022). Several studies have shown that gambling can lead to harmful consequences even during adolescence (De Luigi et al., 2018; Livazović and Bojčić, 2019; Pisarska and Ostaszewski, 2020), such as social impairment, school difficulties, and loss of interest in other activities, as described by Gambling Disorder (GD) criteria proposed by the American Psychiatric Association (APA, 2013). International studies have shown that the percentage of problematic gambling fluctuates between 0.2% and 12.3% in adolescents (Calado et al., 2017).

Consistent with the theoretically hypothesized association, some studies have found a relationship between LB use and problem gambling (e.g., Rockloff et al., 2021). In adult video gamers, LBs opening and spending are associated with problem gambling severity (Zendle and Cairns, 2018; Li et al., 2019; Drummond et al., 2020; Meduna et al., 2020; Hing et al., 2022). Following this kind of evidence, increasing interest has been focused toward the young populations, with similar findings. Zendle et al. (2019) found a positive association—moderate to large in magnitude—between LBs spending and problem gambling among adolescents (16–18 years old). Likewise, another study carried out with 12–16 years-old youth showed a positive correlation between LB engagement and the severity of problem gambling (Kristiansen and Severin, 2020). Moreover, it has been evidenced that the risk of being at-risk/problem gamblers is higher among those adolescents who have purchased or sold items from a LB (Kristiansen and Severin, 2020), indicating that the use of money for LBs transactions may be a risk factor for problematic gambling behavior. Finally, Ide et al. (2021) found that the adolescent gamers who purchased LBs were significantly more likely to exhibit problem gaming, and when controlling for monetary gambling, age, and gender, recent loot box purchasing increased the likelihood of at-risk and problem gambling (Hing et al., 2022).

However, despite some research, relatively few studies have been conducted on LB use in general with adolescents, as highlighted by recent reviews (Gibson et al., 2022; Montiel et al., 2022), and, specifically, on the association between LB use and gambling behavior (Hing et al., 2022), with an open controversy on the nature of the relationship between LB use and problematic gaming and gambling (Montiel et al., 2022). Instead, a better understanding of this link would be essential for more effective interventions both for prevention and treatment. In this work, we advanced and tested the hypothesis that video gaming could be the intermediary variable linking LB use with gambling behavior in adolescents. Our hypothesis is based on an integration of different fields of studies that have, respectively, documented that LB use is associated with video gaming and problem video gaming (e.g., Li et al., 2019; Drummond et al., 2020; Ide et al., 2021; Zendle, 2020), and that the use of video games is in some way related to gambling behavior (e.g., Wood et al., 2004; McBride and Derevensky, 2016; Vadlin et al., 2018; Derevensky and Griffiths, 2019; Molde et al., 2019; Sanders and Williams, 2019; Delfabbro and King, 2020).

Concerning the association of LB use with video gaming, since LBs have video games features and they are embedded into video games, some studies have tried to better understand the relationship that LBs have with video gaming, both in terms of frequency and severity. Overall, these studies observed that LBs spending is associated with video gaming frequency (Li et al., 2019) and the severity of video gaming (Li et al., 2019; Drummond et al., 2020; Zendle, 2020; Ide et al., 2021). Moreover, in a recent meta-analysis, it has been found an average correlation coefficient of 0.26 (CI [0.15, 0.38]) between LBs spending and problem gaming, indicating a potentially relevant association (Garea et al., 2021). Such a relationship is particularly noteworthy if we look at the age group of adolescents, as video gaming is a massively widespread activity among youths. Indeed, in the “new digital age,” in which the technology is easy and simple to access, video games are increasingly available. In recent years, there has been an ever-increasing usage of video games in adolescents and young adults, partly because it is possible to share the gaming experience with thousands of players simultaneously, thanks to new online gaming modes (Balhara et al., 2020; Ponce-Blandón et al., 2020). Specifically, nearly 90% of adolescents are involved in video gaming (Gentile et al., 2011; Gonzálvez et al., 2017). An excessive use of video games can lead to harmful consequences, as described by Internet Gaming Disorder criteria (IGD) proposed by the APA (2013), and with the terms Gaming Disorder in the World Health Organization’s International Classification of Diseases (ICD-11; World Health Organization, 2019). A systematic review indicated that problem video gaming has a mean prevalence of 2% in representative samples of in children and adolescents, and the rates of problem gaming reached 5.5% when the studies have been conducted with clinical samples (Paulus et al., 2018).

With regard to the relationship between the use of video games and gambling behavior, this topic has received increasing interest in the last 30 years, with some studies that have found an overlap between these two behaviors among the juvenile population. McBride and Derevensky (2016) reported that young video game players (16–24 years old) were more likely to be gamblers, compared with the non-players. In addition, gamblers were more likely to be video game players than non-gamblers. Similarly, Wood et al. (2004) found that regular gamblers (i.e., gamblers who played at least once a week) were more likely to be regular video game players as well, compared to non-regular gamblers. Some studies have focused on the relationship between problem gaming and problem gambling (McBride and Derevensky, 2016; Sanders and Williams, 2019). McBride and Derevensky (2016) found that problem gamers had a higher probability of also being problem gamblers compared to social or non-gamers, and Sanders and Williams (2019) evidenced that nearly a tenth of the problem gamblers were also problem video game players. An association between problem gaming and problem gambling was also found in longitudinal studies (Molde et al., 2019; Vladin et al., 2019). Molde et al. (2019), with a sample of 4,601 adolescents, found that problem video gaming predicted problem gambling 2 years later, controlling for the fact that problem gambling was not a predictor of problem video gaming. Vladin et al. (2019) confirmed that problem video gaming predicted gambling 3 years later.

In summary, we know from the literature that: (i) LB use is associated with gambling behavior (e.g., Zendle et al., 2019; Kristiansen and Severin, 2020; Ide et al., 2021); (ii) LB use is also related to video gaming (e.g., Li et al., 2019; Drummond et al., 2020; Zendle, 2020; Ide et al., 2021); and (iii) there is a relationship between video gaming and gambling (e.g., Wood et al., 2004; McBride and Derevensky, 2016; Sanders and Williams, 2019), with longitudinal evidence that video gaming behavior predicts gambling behavior (Molde et al., 2019; Vladin et al., 2019). Following these premises, we hypothesized and tested a model that integrated all the above-cited research lines in order to understand a possible mechanism linking LB use to problem gambling in youth. In detail, we predicted a model in which LB use was the independent variable and problem gambling was the dependent variable. The intermediary role was, respectively, exercised by the use of video games, that was hypothesized to be the most proximal mediator; problem video gaming, that was considered as the less proximal mediator; and gambling frequency, that was thought to be the most distal mediator in the relationship between LB use and problem gambling. The proposed model was supported by a previous study conducted with adult video gamers in which it emerged that LBs purchasing is a predictor of both video gaming frequency and online gambling frequency, which, in turn, were associated, respectively, associated with problem video gaming and problem gambling (Li et al., 2019).

We expected to find that video-gaming behavior would be the mediator between LB use and gambling behavior. In other terms, we predicted that an intense use of LBs would be associated with intense video gaming behavior and, in turn, with a great involvement in gambling and, consequently, with a high level of gambling problem severity. To verify this kind of relationships among the variables, we developed two studies with Italian adolescents. In Study 1, we investigated the adequacy of the predicted model before COVID-19-pandemic-related lockdown. In Study 2, we analyzed the same model in a different historical period, i.e., during COVID-19-pandemic-related lockdown. In this regard, Italy has been the first European country to be affected by the virus in March 2020, and the first to adopt restrictive measures of social distancing. The COVID-19 pandemic led to many changes in people’s daily lives, not only because of the mandatory quarantine and restrictions that involved social estrangement, but also because of the fear of getting infected, which influenced individuals’ mental health (Balhara et al., 2020; Pakpour and Griffiths, 2020). Restrictions were placed on individual freedom of movement from March 9, 2020, as response to the new epidemic emergency, and only essential activities were allowed (Decreto del Presidente del Consiglio dei Ministri, 2020). Not only at a national level, but internationally, enforced restrictions increased the likelihood of addictive behaviors, such as online games, because of the higher time spent at home (Lugo et al., 2021). Referring specifically to youth, school closure and event cancellations during the lockdown have limited adolescents’ social interactions during this pandemic. In the same period, the download and sales rates of apps increased significantly worldwide, and mobile game downloads in Europe reached a record high just in March 2020 (GWI, Global Web Index, 2020; Ko and Yen, 2020; Pacetti and Soriani, 2021; Sultana et al., 2021). Consequently, there has been an enhanced opportunity to use play video games (Guido et al., 2020; Ko and Yen, 2020) and experience problem video gaming symptoms. Longitudinal studies verified the hypothesis of an increase of problem video gaming: In China, it has been found that adolescents increased their video game use during the COVID-19 pandemic, and they experienced significantly increased GD symptoms (Teng et al., 2021); in Germany, Paschke et al. (2021) showed that adolescents’ use of video games significantly increased under the lockdown compared to before the COVID-19 pandemic. Concerning the prevalence of problem gaming during the COVID-19 pandemic, in Hong Kong, there have been found prevalence rates of 20.9% of excessive gamers and 5.3% of pathological gamers in children and adolescents (Zhu et al., 2021), while in Italy, parental evaluations of problem gaming symptoms in their offspring lead to obtain a prevalence of 36% at-risk gamers and 22% problem gamers (Donati et al., 2021). Concerning the use of LBs in adolescents during the pandemic, to the best of our knowledge, we do not have specific information. However, a retrospective English study realized with adults suggested that significant increases in self-reported expenditure on loot boxes have been registered before the pandemic (Close et al., 2022).

As for gambling, among the government’s restriction measures, most gambling activities with physical presence were banned from 12 March to 12 June, 2020 (Agenzie Dogane Monopoli, 2020), as well as internationally. Although little we know about youth’s participation in gambling during the lockdowns, as the populations surveyed to examine gambling during the pandemic to date have involved individuals aged 18 or over (Masaeli and Farhadi, 2021; Riley et al., 2021), a longitudinal study conducted with young adults of about 24 years in the United Kingdom indicated that the overall frequency of gambling reduced during lockdown, while online gambling did increase (Emond et al., 2022).

Thus, given the specificity of the pandemic period, characterized by a high stimulation of video gaming and mostly online gambling, we aimed at take advantage from this particular situation to verify the explanation power of LB use on gambling through video gaming even in times in which youth had potentially more time to game and gamble, especially under the stressful conditions characterizing the pandemic. If Loot Boxes exerted a robust effect, it would have prevailed even during the pandemic. In sum, we hypothesized that both during a usual lifetime for adolescents and during a period of extensive stimulation to Internet and video gaming, an intense use of LBs would lead adolescents to high video gaming behavior and, in turn, to a great involvement in gambling and, consequently, to a high level of gambling problem severity.

## Study 1

Adolescents seem to be increasingly involved in LBs, and research highlights that the familiarity with the LBs is related to video gaming and gambling. However, the mechanisms of these relationships are not clear yet. In response to this gap, the aim of Study 1 was to deeply investigate the relationships between the use of LBs, video gaming behavior, and gambling behavior among adolescents by testing the adequacy of a path model in which LB use was seen as the independent variable, video gaming frequency, problem video gaming, and gambling frequency were considered as mediators, and problem gambling was conceptualized as the outcome variable. We also aimed at having more detailed information about these three behaviors in a wide sample of Italian adolescents. This study was conducted before the beginning of the COVID-19-related pandemic.

### Materials and methods

#### Participants

Participants were 1,078 adolescents (39% boys, *M*age = 16.46, *SD* = 1.28, range: 13–20 years) attending eight public high schools in urban (92%, *n* = 988) and suburban centers in the North East of Italy (Veneto). Specifically, 9% (*n* = 98) of adolescents attended professional training centers, 8% (*n* = 90) of adolescents attended a professional high school, 46% (*n* = 500) of adolescents attended a technical school, and 36% (*n* = 390) a lyceum. High school in Italy consists of 5 years of education. The sample included 10% (*n* = 110) first-year students, 12% (*n* = 132) second-year students, 22% (*n* = 232) third-year students, 45% (*n* = 483) fourth-year students, and 11% (*n* = 121) fifth-year students. The collaboration with schools was part of a larger regional gambling prevention program (Piano Regionale Gioco d’Azzardo Patologico – Regione Veneto). Once the schools agreed to participate, the detailed study protocol was approved by the institutional review boards at each school. Written informed consent was requested from the students’ parents, assuring them that the data would be handled confidentially. The research was conducted at school, during school time, during September–December 2019.

#### Instruments and procedure

Preliminarily, socio-demographic information, such as gender and school, was requested. Age in terms of years and months was also requested.

To assess LB use, the three following questions were administered to high school students: (1) *Have you found a loot box during your video gaming sessions*?; (2) *Have you opened a loot box during your video gaming sessions*?; (3) *Have you bought a loot box, or a key to unlock it, during your video gaming sessions*? In order to have a quantitative measure of LB use, in the scoring phase, an LB use index was computed by summing the responses (No = 0; Yes = 1) to the above-reported questions.

To investigate video gaming behavior, the *Video Gaming Scale – For Adolescents* (VGS-A; Primi et al., 2017) was employed. The instrument is divided into two sections: the first is related to video gaming habits, while the second is intended to investigate symptoms of pathological video gaming based on the DSM-5 (APA, 2013). Both sections referred to the last 12 months. More specifically, in the first section, adolescents were asked to report as to whether they had used video games during that period [*yes*, *no*]. Based on the response, adolescents were classified as video gamers/non-video gamers. They were also asked to indicate the frequency with which (*never* = 0, *sometimes* = 1, *often* = 2) they engaged in a list of 16 game genres, based on a previous game genre classification (Donati et al., 2015). By summing responses given each game genre, a total score indicative of video gaming frequency was obtained. Moreover, adolescents had to declare indicate how many hours a day and how many days a week they spent by gaming on the listed devices (console, computer, smartphone, tablet). In order to determine time spent on VGs *per* device, we multiplied the hours per days. The first section responds to descriptive objectives and does not contribute to the calculation of the problematic behavior score. The second section of the VGS-A is composed of nine items, each one developed in order to reveal one of the nine symptoms listed in DSM-5 concerning pathological video gaming, provided on a 3-point Likert scale: *never* (0), *few times* (1), and *many times* (2). The advantage of using this instrument is that the scoring system has been developed by applying Item Response Theory (IRT), in order to have a measure of problem gaming that took into account the severity of each symptom described by the items. Thus, the total score represents a weighted score based on the specificity of the items endorsed (in terms of its own severity and discrimination power) and the response endorsed (few times or many times). The IRT-based score allows for the classification of adolescents into non-problem, at-risk, and problem video gamers. The VGS-A is an efficient tool to assess mid-to-high levels of the DSM-5 Gaming Disorder criteria in adolescents.

To measure gambling behavior, we administered the *Gambling Behaviour Scale for Adolescents* (GBS-A; Primi et al., 2015), which has a similar structure with respect of the VGS-A. Indeed, the GBS-A is composed of two sections, too, both referred to the last 12 months. The first section consists of unscored items investigating gambling frequency. In detail, 10 items assess the frequency (*never*, *sometimes in the year*, *sometimes in the month*, *sometimes in the week*, *daily*) of participation during the last year in ten gambling activities (card games, bets on games of personal skill, bets on sports games, bets on horse races, bingo, slot machines, scratch cards, lotteries, online games, and private bets with friends). Based on their responses to this section, participants were identified as non-gamblers (no gambling behavior) or gamblers (gambling on at least one activity; Welte et al., 2009), and gamblers can be classified into non-regular gamblers (those adolescents who gamble less frequently than weekly) and regular gamblers (those adolescents who gamble daily or weekly on at least one gambling activity; Winters et al., 1993). The second section consists of nine scored items assessing the DSM-5 (APA, 2013) diagnostic criteria for Gambling Disorder. Each item is evaluated on a three-point Likert scale ranging from *Never* (0) to *Often* (2). Based on the responses to this section, it is possible to derive an Item Response Theory-based score for each respondent. Following this IRT-based scoring procedure, respondents can be classified into non-problem gamblers, at-risk gamblers, and disordered gamblers. The GBS-A has been shown to be unidimensional and useful for mid-to-high levels of Gambling Disorder severity (Donati et al., 2017).

The above-described scales were administered in the classrooms and students were required to work individually. The order of presentation was the following: GBS-A, VGS-A, and the questions about the LB use. Administration of the instruments was realized by the staff of the “Gambling team” of the Addictions Department AULSS4 Veneto Orientale and it required ~50 min.

### Results

#### Loot boxes use, video gaming, and gambling description

Most of the participants were familiar with the LBs. Over the last 12 months, 76% (*n* = 814) of the participants found at least one LB during video game playing sessions, 73% (*n* = 791) opened it, and 25% (*n* = 268) purchased it. By summing responses to the three items, the obtained composite score, i.e., the LB use, had an average value of 2.29 (*SD* = 0.53).

Concerning video gaming, most of the participants (91%; *n* = 986) declared to play video games in the last 12 months. Video gamers had a mean age of 16.47 years (*SD* = 1.27; range: 13–20) and 65% (*n* = 639) of them were males. The most practiced game genres were Action games, followed by First Person Shooter games, Real Time Strategy games, and Sport games. Table 1 reports the prevalence of video gamers for each video game genre.

**Table 1:** Prevalence of video gamers at each video game genre in Study 1 (*n* = 996).

Video game genres	Video gamers % (*n*)
Action	71% (708)
Real-time strategy	66% (655)
First person shooter	65% (650)
Role-playing	56% (559)
Management	54% (535)
Simulation	49% (492)
Party	44% (441)
Platform	42% (576)
Adventure	38% (380)
Casual	36% (358)
Fighting	36% (363)
Sports	35% (647)
Puzzle games	34% (280)
Massively multiplayer online (MMO)	32% (318)
Sandbox	29% (294)
Multiplayer online battle arena (MOBA)	25% (249)

Adolescents reported to play video games mostly by using smartphones (*M*hours*per*day = 7.40, *SD* = 10.64). Console (*M*hours*per*day = 3.64, *SD* = 7.07), computer (*M*hours*per*day = 2.66, *SD* = 7.44), and tablet (*M*hours*per*day = 1.13, *SD* = 4.19) were less used.

Regarding problem gaming, almost three-quarter of the video gamers (72%, *n* = 708) were non-problem video gamers, while 23% (*n* = 223) were at-risk video gamers, and 5% (*n* = 54) were problem video gamers.

With respect to gambling, findings showed that 71% (*n* = 761) of the participants gambled on at least one gambling activity over the last 12 months. Gamblers had a mean age of 16.49 years (*SD* = 1.27; range: 13–20) and 67% of them (*n* = 510) were males. Among the gamblers, 25% (*n* = 194) were regular gamblers. The most engaged activities resulted to be Scratch cards, Bingo, and Cards for money. The prevalence of gamblers on each gambling activity is reported in Table 2.

**Table 2:** Prevalence of gamblers on each gambling activity in Study 1 (*n* = 761).

Gambling activity	Gamblers % (*n*)
Scratch cards	52% (396)
Bingo	49% (374)
Card games	44% (339)
Private bets with friends	42% (314)
Bets on sport games	36% (274)
Bets on games of personal skills	35% (266)
Online gambling activities	23% (179)
Online gambling activities	23% (179)
Lotteries	16% (362)
Slot machines	10% (75)
Bet on horse races	4% (29)

As concerns problem gambling, 88% (*n* = 66) of the gamblers were non-problem gamblers, 10% (*n* = 73) were at-risk gamblers, and 2% (*n* = 20) were problem gamblers.

#### Path model about the relationships among Loot boxes use, video gaming, and gambling

As a first step, bivariate correlations among LB uses, video gaming frequency, problem video gaming, gambling frequency, and problem gambling, were computed. We found that LB use correlated significantly and positively both with video gaming frequency and problem video gaming, and with gambling frequency and problem gambling. In detail, the correlations were mild–moderate in size. Significant, positive, and large size correlations were found between video gaming frequency and problem gaming, and between gambling frequency and problem gambling. Moreover, gaming and gambling behaviors resulted to be moderately correlated (Table 3).

**Table 3:** Means, standard deviations, and correlations among LB use, video gaming frequency, problem video gaming, gambling frequency, and problem gambling, in Study 1.

Variables	1	2	3	4	5
1. LB use	–				
2. Video gaming frequency	0.35^***^	–			
3. Problem video gaming	0.34^***^	0.51^***^	–		
4. Gambling frequency	0.25^***^	0.32^***^	0.24^***^	–	
5. Problem gambling	0.23^***^	0.24^***^	0.46^***^	0.43^***^	–
*M*	2.29	9.19	4.42	3.38	1.41
SD	0.53	6.23	3.61	4.03	2.18

LB, Loot Box.

*** *p* < 0.001.

To test our hypothesis about the relationships between the LB use, gaming, and gambling, we conducted a path analysis with AMOS 16 software (IBM SPSS Statistics, Armonk, NY, United States; Arbuckle, 2007) using maximum likelihood estimation. The presence of meditated effects among the variables was investigated through the test of indirect effects (Cheung and Lau, 2008). In AMOS, the bootstrap confidence interval method to define the confidence intervals for indirect effects (MacKinnon et al., 2004) was implemented. In mediation analysis, bootstrapping is used to generate an empirically derived representation of the sampling distribution of the indirect effect, and this empirical representation is used for the construction of a confidence interval for the indirect effect. The 90% bias-corrected confidence interval percentile method was implemented using 2000 bootstrap samples. Confidence intervals for the indirect effects, which do not contain 0, are considered indicative of significant indirect effects, thus meaning the presence of a mediated effect. Several goodness-of-fit indices were used to test the adequacy of the model: the comparative fit index (CFI; Bentler, 1990), the Tucker–Lewis index (TLI; Tucker and Lewis, 1973), and the Root Mean Square Error of Approximation (RMSEA; Steiger and Lind, 1980). CFI and TLI values equal to 0.90 or greater (Tucker and Lewis, 1973; Bentler, 1990) and RMSEA values of.08 or below (Steiger and Lind, 1980) are considered indices of adequate fit.

**Figure 1:**
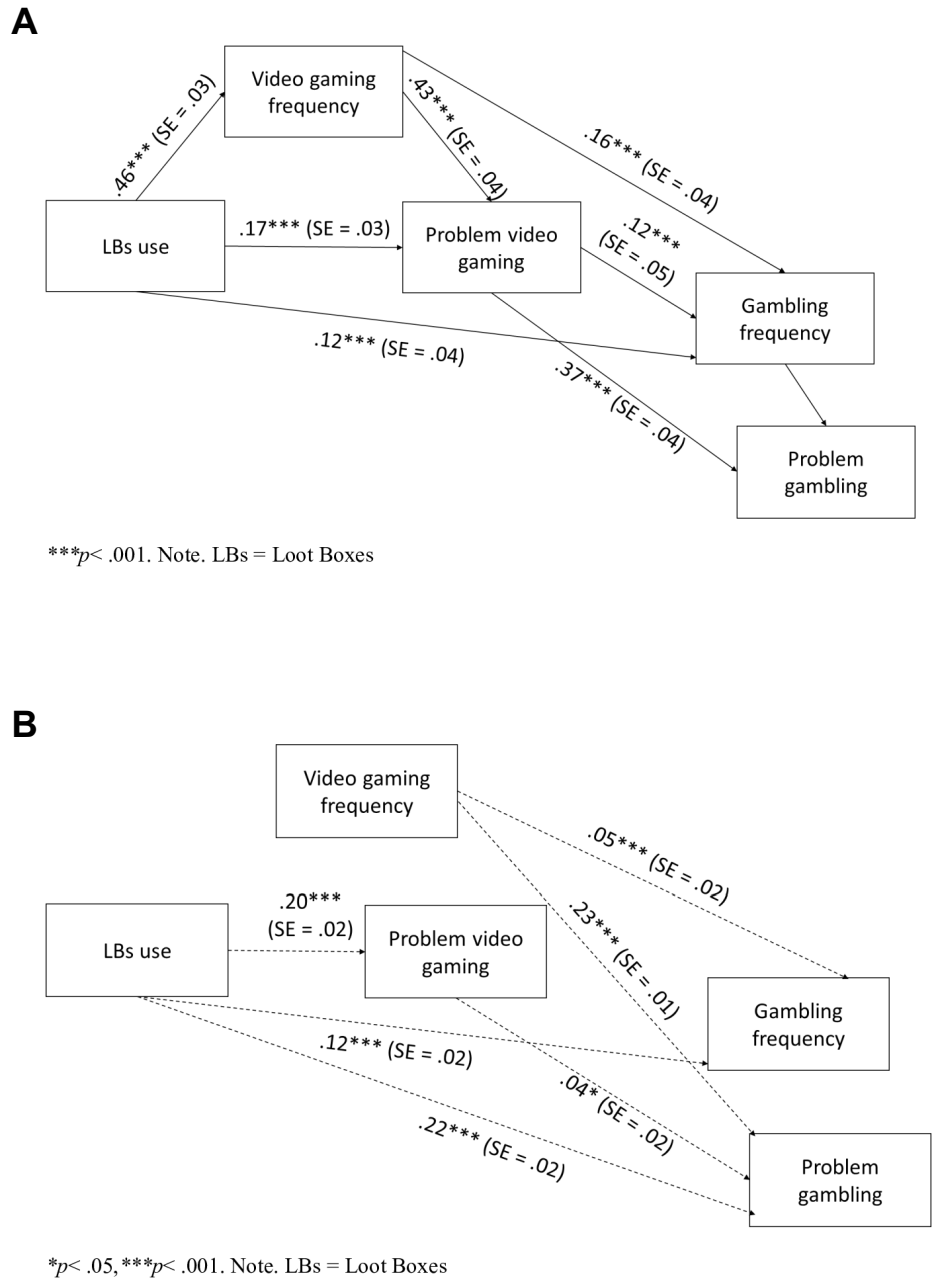
**(A)** Path model with direct effects among the variables in Study 1. **(B)** Path model with indirect effects among the variables in Study 1.

Results showed that the goodness-of-fit indices of the proposed model were indicative of a good fit (CFI = 0.995, TLI = 0.976, RMSEA = 0.05). As hypothesized, LB use had a significant and positive direct effect on video gaming frequency, problem video gaming, and gambling frequency. Moreover, video gaming frequency exerted a significant and positive direct effect on problem video gaming and gambling frequency, which, in turn, had a positive direct effect on problem gambling (Figure 1A).

Results also showed significant and positive indirect effects: (i) from LB use to problem video gaming, passing by video gaming frequency; (ii) from LB use to gambling frequency through problem video gaming; and (iii) from LB use to problem gambling through problem video gaming. Moreover, video gaming frequency had significant positive indirect effects on gambling frequency and problem gambling with the intermediary role of problem video gaming, and problem video gaming exerted a significant and positive indirect effect on problem gambling through gambling frequency (Figure 1B).

### Discussion

The aim of Study 1 was to investigate the relationship between the use of LBs, video gaming behavior, and gambling behavior among adolescents. Secondary objectives regarded the analysis of these behaviors in a wide sample of adolescents.

Findings attest that the majority of the sample knows LBs and one out of four students purchased them while playing video games. Our data confirm and extend the previous scan research studies with adolescents (Zendle et al., 2019; Kristiansen and Severin, 2020; DeCamp, 2021; Ide et al., 2021). Consistent with previous studies, we found that the large majority of adolescents played video games in the last year (Kowert et al., 2014; Müller et al., 2015; Rehbein et al., 2015; Pontes et al., 2016), especially Action games, First Person Shooter games, Real Time Strategy games, and Sport games (López-Fernández et al., 2020), and mainly by using the smartphone (Lemola et al., 2015). Moreover, the percentages of video gamers classified as at-risk and problem gamers are comparable with what emerged in times before the COVID-19-related pandemic (Gentile, 2009; Thomas and Martin, 2010; Rehbein et al., 2015; Milani et al., 2018). Concerning gambling, most of the participants gambled on at least one gambling activity over the last 12 months (Calado et al., 2017; Donati et al., 2022), and about a quarter gambled on a regular basis, in line with literature (Hollén et al., 2020; Tani et al., 2021). The most engaged activities resulted to be Scratch cards, Bingo, and Cards for money (Calado et al., 2017; Dowling et al., 2017;; Donati et al., 2022). Among the gamblers, the prevalence of at-risk and problem gamblers was consistent with the international prevalence rates (Calado et al., 2017).

In response to the necessity to better investigate the relationship between LB use, video gaming, and gambling (Gibson et al., 2022; Montiel et al., 2022), our findings indicate that LB use (that comprehends having found a loot box, having opened it, and having bought it) affects problem gambling through the intermediary role of video gaming frequency and problem video gaming, and gambling frequency. That means that as adolescents become more familiar with the LBs, they become more at risk of developing symptoms of problem gaming by spending more time on the video games. Through this increased use of video games, adolescents may become more involved in gambling and, consequently, they are more likely to encounter symptoms of Gambling Disorder. Overall, this model underlines that LBs can be particularly harmful for the young video game players.

## Study 2

To verify if the set of relationships among LB use, video gaming, and gambling in adolescents found in Study 1 maintained also during the pandemic lockdown, a period of extensive stimulation to Internet and video gaming, we also conducted a study during COVID-19-pandemic-related lockdown. Indeed, both internationally and nationally, in this period, there has been an increase of video gaming behavior among the younger generations (Guido et al., 2020; Ko and Yen, 2020) with enhanced likelihood of developing problem video gaming symptoms (Donati et al., 2021; Paschke et al., 2021).

### Materials and methods

#### Participants

One thousand two hundred and four adolescents (57% boys, *M*age = 16.10, *SD* = 0.65, range, 15–19 years) attended 26 public high schools in urban and suburban centers in the Center of Italy (Tuscany). In detail, 5% (*n* = 65) of adolescents attended professional training centers, 16% (*n* = 195) of adolescents attended a professional high school, 33% (*n* = 398) of adolescents attended a technical school, and 45% (*n* = 546) attended a lyceum. All the students were attending the second year of high school. In detail, 41% of the schools were located in the center of Tuscany, 21% of the schools were in the North-West of the Region, and 38% of the schools were placed in the South-East of Tuscany.

This study took part of a larger regional gambling prevention program (PRIZE [Prevention of gambling risks among adolescents], Resolution of the Tuscany Region n. 771, 9 July 2018), in which the issue of video gaming and micro-economic transitions inside video games was investigated. The project was approved by the institutional review boards at each school. Written informed consent was requested from the students’ parents. As the study was conducted during May–June 2020, period in which the Italian high schools were under the remote teaching regime, the research was conducted during school time but through an online administration. In other terms, students were at their own homes, and they completed the research protocol through an online platform.

#### Instruments and procedure

The instruments used were the same employed in Study 1. Socio-demographic information, such as gender, age, and school, was still requested.

LB use was assessed through the same three questions. However, to have a response related to the period of the COVID-19-related pandemic, the questions were explicitly referred to the last 12 months, instead of the last 12 months, as in Study 1. By summing the responses (No = 0; Yes = 1), we obtained a measure of LB use.

The VGS-A (Primi et al., 2017) was used to measure video gaming behavior features (Section I), and related pathological gaming behavior symptoms according to the DSM-5 (Section II). Through the second section, both a quantitative measure and a categorical variable regarding the severity of video gaming behavior can be drawn. In order to collect information about gaming habits referred to the specific period of the lockdown, the VGS-A was framed in relation to the last 2 months.

The GBS-A (Primi et al., 2015) was employed to assess gambling behavior habits and related severity by referring to the last 2 months in this study. Consequently, the response scale of items investigating gambling frequency at a series of gambling activities was: *never*, *once in the month*, *sometimes in the month*, *sometimes in the week*, and *daily*. Overall, with the first section, we investigated the presence of a gambling behavior, gambling frequency by gambling activity, and in general, as well as the presence of a regular gambling behavior. Through the second section, consisting of items assessing the DSM-5 diagnostic criteria for Gambling Disorder, we derived a quantitative measure and a categorical variable about the severity of gambling behavior.

The above-described scales were administered through an online link with a Google Forms. Students were at home. They were required to work individually. The order of presentation was the following: GBS-A, VGS-A, and the questions about the LB use. Administration of the instruments was realized by the intervention providers of the PRIZE project, and it required ~40 min.

### Results

#### Loot boxes use, video gaming, and gambling description

More than half of the sample had some experience with the LBs. Specifically, 54% (*n* = 623) of adolescents encountered at least one loot box playing video games, 52% (*n* = 605) opened it, and 9% (*n* = 110) purchased it. The mean score of the LBU was 1.16 (*SD* = 1.09).

Most of the participants (90%; *n* = 1,064) resulted to play video games. Video gamers had a mean age of 15.56 years (*SD* = 0.57; range: 14–18), and 62% (*n* = 657) of them were males. The most practiced game genres were First Person Shooter games, followed by Action games and Sport games. Table 4 reports the prevalence of video gamers for each video game genre.

**Table 4:** Prevalence of video gamers at each video game genre in Study 2 (*n* = 1,064).

Video game genre	Video gamers % (*n*)
First person shooter	64% (679)
Action	54% (570)
Sports	47% (498)
Casual	36% (381)
Sandbox	35% (369)
Management	33% (350)
Party	29% (303)
Platform	28% (301)
Simulation	20% (214)
Fighting	16% (165)
Puzzle games	16% (170)
Multiplayer online battle arena (MOBA)	15% (153)
Real-time strategy	15% (162)
Role-playing	10% (107)
Massively multiplayer online (MMO)	9% (97)
Adventure	9% (91)

The most used device to play video games was the smartphone (*M*hours*per*day = 4.12, *SD* = 4.54). Console (*M*hours*per*day = 2.01, *SD* = 2.60), computer (*M*hours*per*day = 1.65, *SD* = 2.82), tablet (*M*hours*per*day = 0.51, *SD* = 1.72), and handheld console (*M*hours*per*day = 0.22, *SD* = 1.18) were less used.

The majority of the video gamers (78%, *n* = 811) were non-problem video gamers, 19% (*n* = 194) were at-risk video gamers, and 3% (*n* = 31) were problem video gamers.

Concerning gambling, 60% (*n* = 712) of the participants gambled on at least one gambling activity. Gamblers had a mean age of 15.62 years (*SD* = 0.64; range: 15–19) and 61% of them (*n* = 434) were males. Among the gamblers, 25% (*n* = 176) were regular gamblers. The most engaged activities resulted to be Online gambling activities, Scratch cards, and Bingo. The prevalence of gamblers on each gambling activity is reported in Table 5.

**Table 5:** Prevalence of gamblers on each gambling activity in Study 2 (*n* = 712).

Gambling activities	Gamblers % (*n*)
Online gambling activities	53% (376)
Scratch cards	41% (288)
Bingo	33% (237)
Private bets with friends	27% (190)
Bets on sport games	23% (164)
Bets on games of personal skills	20% (145)
Card games	19% (136)
Lotteries	9% (63)
Slot machines	6% (43)
Bet on horse races	4% (34)

Eighty-four percent of the gamblers (*n* = 579) were non-problem gamblers, 11% (*n* = 78) were at-risk gamblers, and 5% (*n* = 35) were problem gamblers.

#### Path model about the relationships among LBU, video gaming, and gambling

From the correlation matrix, we found that LBU correlated significantly and positively with all the variables related to video gaming and gambling. In detail, the correlations were moderate in size with video gaming frequency and problem gaming, and large in size with gambling frequency and problem gambling. Significant, positive, and large size correlations were found between video gaming frequency and problem gaming, and between gambling frequency and problem gambling. Moreover, gaming and gambling behaviors resulted to be moderately correlated (Table 6).

**Table 6:** Means, standard deviations, and correlations among LB use, video gaming frequency, problem video gaming, gambling frequency, and problem gambling, in Study 2.

Variables	1	2	3	4	5
1. LB use	–				
2. Video gaming frequency	0.44^***^	–			
3. Problem video gaming	0.38^***^	0.49^***^	–		
4. Gambling frequency	0.23^***^	0.35^***^	0.30^***^	–	
5. Problem gambling	0.15^***^	0.29^***^	0.45^***^	0.47^***^	–
*M*	1.16	5.25	3.21	2.20	0.98
SD	1.09	4.44	3.36	2.97	2.16

LB, Loot Box.

*** *p* < 0.001.

**Figure 2:**
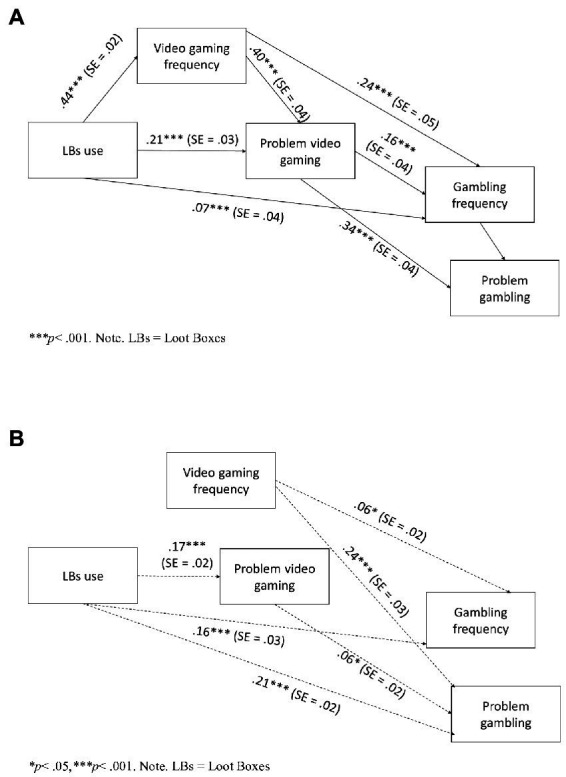
**(A)** Path model with direct effects among the variables in Study 6. **(B)** Path model with indirect effects among the variables in Study 2.

To test the model verified in Study 1, we conducted again a path analysis with AMOS 16 software (IBM SPSS Statistics, Armonk, NY, United States; Arbuckle, 2007) using maximum likelihood estimation, and we obtained a good fit (CFI = 0.994, TLI = 0.97, RMSEA = 0.06). In detail, significant and positive direct effects were found: (i) from LBU to video gaming frequency, problem video gaming, and gambling frequency; (ii) from video gaming frequency to problem video gaming and gambling frequency; and (iii) from gambling frequency to problem gambling (Figure 2A).

We also found significant and positive indirect effects: (i) from LBU to problem video gaming, passing by video gaming frequency; (ii) from LBU to gambling frequency through problem video gaming; (iii) from LBU to problem gambling through problem video gaming; (iv) from video gaming frequency to gambling frequency and problem gambling with the intermediary role of problem video gaming; and (v) from problem video gaming to problem gambling through gambling frequency (Figure 2B).

### Discussion

In Study 2, we aimed at verifying if the set of relationships among LB use, video gaming, and gambling in adolescents found in Study 1, i.e., before the COVID-19-related pandemic, was still adequate considering the high stimulation of the Internet and video games that occurred during the COVID-10 pandemic. First, concerning the prevalence of the behaviors under attention in this work during this specific time frame, we found that more than half of the sample knows LBs and 9 % of the students purchased them while playing video games. The large majority of adolescents played video games, especially, First Person Shooter games, Action games, and Sport games, and mainly by using the smartphone. As for gambling. 60% of the participants gambled on at least one gambling activity, with a quarter of them that was regular gamblers. The most engaged activities resulted to be Online gambling games, Scratch cards, and Bingo. With respect to the COVID-19-specific research studies, the prevalence of video gamers is in line with data available (Donati et al., 2021; Teng et al., 2021), as well as the prevalence of video gamers based on adolescents’ self-evaluations (Zhu et al., 2021). Moreover, consistent with a previous published study with young adults (Emond et al., 2022), we found that adolescents were mainly involved in online gambling activities.

Findings of Study 2 indicated that the path model verified in Study 1 concerning the relationships between LB use and problem gambling through video gaming frequency, problem video gaming, and gambling frequency was still adequate in time of pandemic. Comparable coefficients with respect to Study 1 were found for direct and indirect effects. Thus, we supported our hypothesis that, even in a period of increased invitations and stimulation of using the Internet and video games, higher use and familiarity with the LBs makes adolescents more at risk of developing symptoms of problem gaming by spending more time on the video games. As a consequence, youth are at risk of becoming more involved in gambling and to develop pathological gambling.

## General discussion

Two recent reviews (Gibson et al., 2022; Montiel et al., 2022) clearly indicated that relatively scan research currently exists about LB use in adolescents. Moreover, the association between LB use and gambling behavior is still not clear (Hing et al., 2022), especially the relationship between LB use and problematic gaming and gambling (Montiel et al., 2022). Based on an integration of previous research findings indicating that LB use is associated with video gaming and problem video gaming (e.g., Li et al., 2019; Drummond et al., 2020; Zendle, 2020; Ide et al., 2021), and that the use of video games is related to gambling behavior (e.g., Wood et al., 2004; McBride and Derevensky, 2016; Vadlin et al., 2018; Derevensky and Griffiths, 2019; Molde et al., 2019; Sanders and Williams, 2019; Delfabbro and King, 2020), we advanced the hypothesis that video gaming could be the intermediary variable linking LB use with problem gambling in adolescents. Our mediation model was confirmed through two studies, respectively, conducted before and during the COVID-19-related pandemic. More specifically, we demonstrated that a high use of LBs may make adolescents more at risk of developing symptoms of problem gaming by spending more time on the video games. As a consequence, youth are at risk of becoming more involved in gambling and to develop pathological gambling. This path of relationships occurred both in pre-pandemic times, during which adolescents could spend their times inside and outside home, and during the pandemic lockdown, when youth have been restricted in their possibilities to exit from home, and they passed more time on the Internet, on which they increased their time both on video games and on gambling activities.

Thus, this work supports that there can be a specific pathway that originates from the loot boxes and, through a greater involvement in video games, contributes to the risk for problem gambling. Our findings are part of and carry forward the previous studies conducted in the field of research on the relationship between gaming and gambling. In this regard, nearly 30 years ago, it has been noted for the first time that video games and certain gambling activities (i.e., Slot machines, video lottery terminals) had similar structural characteristics (Griffiths, 1991). For example, both activities take advantage of the variable ratio schedule of reinforcement, and they increase the physiological arousal through lights and sounds (McBride and Derevensky, 2016). In addition, in both the activities, there is a chance of winning something: money or prizes in the case of gambling, and points in the case of video games (Griffiths, 1991). Despite the similarities, one of the main differences between gaming and slot machines is about the outcome, which is predominantly determined by chance in gambling and by the gamers’ ability in video games. For these reasons, it has been proposed that gaming could represent a non-financial form of gambling (Griffiths, 1991). Since 1991, the boundaries between video games and gambling have become increasingly blurred. Nowadays, gambling and gaming activities are available on the same platform. For example, it has become possible to gamble or play video games with the same device (e.g., mobile phone, personal computer). With the introduction of the loot boxes in recent years, video games are increasingly become “*gamblified*”; in turn, gambling activities have increasingly adopted some features of gaming (Gainsbury et al., 2015; Brock and Johnson, 2021), creating the so-called phenomenon of the digital convergence (Griffiths et al., 2013; Gainsbury et al., 2015). Following these research field, it could be that a specific subtype of adolescents classified as problem gamblers are the result of the pathway developing from an intense LB use and a great video gaming behavior.

## Conclusion

The results provide important implications from a practical perspective in terms of prevention and treatment. For prevention, although several preventive approaches exist in literature (King et al., 2019), currently there are no studies addressed to work specifically on the potential relationship between video games and gambling, particularly emphasizing the risks associated with the LBs. Psychoeducational activities should be organized to teach adolescents which are the main features of gambling in order to recognize some of them in the LBs, and to make adolescents more aware of the nature of these video game ingredients. Parental training activities should be also planned as parental monitoring acts as a protective factor against the development of pathological gaming (Chiu et al., 2004; Su et al., 2018). During the COVID-19-pandemic-related lockdown, the gaming use and problematic gaming have considerably increased (Guido et al., 2020; Ko and Yen, 2020; Donati et al., 2021; Paschke et al., 2021; Teng et al., 2021; Zhu et al., 2021; Volpe et al., 2022). After the lockdown period, the requests for professional help for Gaming Disorder to the National Health System are augmented in Italy (Istituto Superiore di Sanità, study in progress). This study clearly suggests that, during the gaming assessment, the clinician should also investigate about the use of LBs in order to carry out an early identification of eventual Gambling Disorder symptoms. It is crucial to assess into the opening, purchase (yes/no), frequency, number of loot boxes and/or loot box expenditure, and use across various time frames to identify the eventual presence of gambling beliefs and problematic gambling behavior. This clinical assessment should also allow for an early identification of Gambling Disorder symptoms, that allows for timely intervention and increases the likelihood of successful recovery and minimizes harms.

Political reflections can be drawn from the present work, in line with what is supported by other researchers (Drummond et al., 2019; Zendle et al., 2019). Being LBs full of gambling similar elements and being a risk factor for problem gaming and problem gambling, political legislator should question about the necessity of regulating these “veiled forms of gambling” (Li et al., 2019, p. 33), as currently happens in some countries like Netherlands, Belgium, China, and Denmark have adopted regulations to limit or bar certain features of loot box that highly resemble gambling. Some concrete action for harm minimization should be realized. In this perspective, in order to better understand the impact of the LBs on the adolescent lives, it should be important to include the assessment of the use of LBs (see Montiel et al., 2022, for a review on the operationalization of loot boxes) into national and international epidemiological surveys with adolescents. With this goal, the three questions used in the present work should allow researchers to briefly and adequately measure different aspects of the familiarity with the LBs.

Some limitations characterized this work. First, both the studies have a correlational nature. The cross-sectional design does not allow us to establish temporal and causal relationships between the variables. Moreover, although the role of the various variables in the path model (i.e., independent variable, mediators, dependent variable) was based on previous research findings, future longitudinal studies should be important to give robustness to our findings. Second, although the same instruments have been used in Study 1 and Study 2, the pandemic does not allow for a traditional paper and pencil in-person administration of the scales. Thus, there is not a completely equal situation in terms of assessment across the two studies. Future studies should further support and extend our model, for instance by including in the model other relevant variables. In this regard, as Brooks and Clark (2019) have pointed out that cognitive distortions correlate with problematic aspects of loot box use, indicating that cognitive distortions can be risk factors for loot box engagement, future research should focus on the construct of erroneous cognitions as an additional linking variable from loot boxes to gambling. Moreover, it should be useful to also assess the risky use of LBs instead of solely the use. Finally, some important psychological dimensions related to problem video gaming and problem gambling, especially personality traits as impulsivity and sensation seeking, should be were not included in this work. Future studies should consider also individual differences correlated to problem video gaming and problem gambling, in order to better understand the personality characteristics typical of the subtype of young problem gambler that originates from the use of LBs.

## Data availability statement

The raw data supporting the conclusions of this article will be made available by the authors, without undue reservation.

## Ethics statement

The studies involving human participants were reviewed and approved by the Schools’ Committees. Written informed consent to participate in this study was provided by the participants' legal guardian/next of kin.

## Author contributions

CP and MD: conceptualization. FS, MV, and MD: data curation and investigation, methodology, and writing—original draft. MD: statistical analysis. CP and ES: supervision and writing—review and editing. All authors contributed to the article and approved the submitted version.

## Funding

Study 1 was funded by the Veneto Region connected to the funding assigned by DGR n. 749, 28 May 2018. The regional funding concerns the prevention and contrast of pathological gambling. Study 2 was funded by the Tuscany Region (Resolution of the Tuscany Region n. 771, 9 July 2018) through ANCI TOSCANA (Association of Tuscan Municipalities).

## Conflict of interest

The authors declare that the research was conducted in the absence of any commercial or financial relationships that could be construed as a potential conflict of interest.

## Publisher’s note

All claims expressed in this article are solely those of the authors and do not necessarily represent those of their affiliated organizations, or those of the publisher, the editors and the reviewers. Any product that may be evaluated in this article, or claim that may be made by its manufacturer, is not guaranteed or endorsed by the publisher.
