# Supplementary material for: Loot boxes use, video gaming, and gambling in adolescents: Results from a path analysis before and during COVID-19-pandemic-related lockdown in Italy
Source: Front Psychol. 2022 Sep 27;13:1009129. doi: 10.3389/fpsyg.2022.1009129 (PMC9551606; doi:10.3389/fpsyg.2022.1009129)
Supplement: Supplementary file 1 [file Data_Sheet_1.docx]

Table 1

*Prevalence of video gamers at each video game genre in Study 1 (n = 996).*

| *Video game genres* | *Video gamers % (n)* |
| --- | --- |
| Action | 71% (708) |
| Real time strategy | 66% (655) |
| First Person Shooter | 65% (650) |
| Role playing | 56% (559) |
| Management | 54% (535) |
| Simulation | 49% (492) |
| Party | 44% (441) |
| Platform | 42% (576) |
| Adventure | 38% (380) |
| Casual | 36% (358) |
| Fighting | 36% (363) |
| Sports | 35% (647) |
| Puzzle games | 34% (280) |
| Massively multiplayer online (MMO) | 32% (318) |
| Sandbox | 29% (294) |
| Multiplayer Online Battle Arena (MOBA) | 25% (249) |

Table 2

*Prevalence of gamblers on each gambling activity in Study 1 (n = 761).*

| *Gambling activity* | *Gamblers % (n)* |
| --- | --- |
| Scratch cards | 52% (396) |
| Bingo | 49% (374) |
| Card Games | 44% (339) |
| Private bets with friends | 42% (314) |
| Bets on sport games | 36% (274) |
| Bets on games of personal skills | 35% (266) |
| Online gambling activities | 23% (179) |
| Online gambling activities | 23% (179) |
| Lotteries | 16% (362) |
| Slot machines | 10% (75) |
| Bet on horse races | 4% (29) |

Table 3

*Means, standard deviations, and correlations among* *LBs use, video gaming frequency, problem video gaming, gambling frequency, and problem gambling, in Study 1.*

| Variables | 1. | 2. | 3. | 4. | 5. |
| --- | --- | --- | --- | --- | --- |
| 1. LBs use | - |  |  |  |  |
| 1. Video gaming frequency | .35*** | - |  |  |  |
| 1. Problem video gaming | .34*** | .51*** | - |  |  |
| 1. Gambling frequency | .25*** | .32*** | .24*** | - |  |
| 1. Problem gambling | .23*** | .24*** | .46*** | .43*** | - |
| *M* | 2.29 | 9.19 | 4.42 | 3.38 | 1.41 |
| *SD* | .53 | 6.23 | 3.61 | 4.03 | 2.18 |

Note. LB = Loot Box.

****p* < .001

Table 4

*Prevalence of video gamers at each video game genre in Study 2 (n = 1064).*

| *Video game genre* | *Video gamers % (n)* |
| --- | --- |
| First Person Shooter | 64% (679) |
| Action | 54% (570) |
| Sports | 47% (498) |
| Casual | 36% (381) |
| Sandbox | 35% (369) |
| Management | 33% (350) |
| Party | 29% (303) |
| Platform | 28% (301) |
| Simulation | 20% (214) |
| Fighting | 16% (165) |
| Puzzle games | 16% (170) |
| Multiplayer Online Battle Arena (MOBA) | 15% (153) |
| Real time strategy | 15% (162) |
| Role playing | 10% (107) |
| Massively multiplayer online (MMO) | 9% (97) |
| Adventure | 9% (91) |

Table 5

*Prevalence of gamblers on each gambling activity in Study 2 (n = 712).*

| *Gambling activities* | *Gamblers % (n)* |
| --- | --- |
| Online gambling activities | 53% (376) |
| Scratch cards | 41% (288) |
| Bingo | 33% (237) |
| Private bets with friends | 27% (190) |
| Bets on sport games | 23% (164) |
| Bets on games of personal skills | 20% (145) |
| Card Games | 19% (136) |
| Lotteries | 9% (63) |
| Slot machines | 6% (43) |
| Bet on horse races | 4% (34) |

Table 6

*Means, standard deviations, and correlations among* *LBs use, video gaming frequency, problem video gaming, gambling frequency, and problem gambling, in Study 2.*

| Variables | 1. | 2. | 3. | 4. | 5. |
| --- | --- | --- | --- | --- | --- |
| 1. LBs use | - |  |  |  |  |
| 1. Video gaming frequency | .44*** | - |  |  |  |
| 1. Problem video gaming | .38*** | .49*** | - |  |  |
| 1. Gambling frequency | .23*** | .35*** | .30*** | - |  |
| 1. Problem gambling | .15*** | .29*** | .45*** | .47*** | - |
| *M* | 1.16 | 5.25 | 3.21 | 2.20 | .98 |
| *SD* | 1.09 | 4.44 | 3.36 | 2.97 | 2.16 |

Note. LB = Loot Box.

****p*<.001
